# “The Heart Game”: Using Gamification as Part of a Telerehabilitation Program for Heart Patients

**DOI:** 10.1089/g4h.2015.0001

**Published:** 2016-02-01

**Authors:** Marcus Dithmer, Jack Ord Rasmussen, Erik Grönvall, Helle Spindler, John Hansen, Gitte Nielsen, Stine Bæk Sørensen, Birthe Dinesen

**Affiliations:** ^1^ Department of Computer Science, Aarhus University, Aarhus, Denmark.; ^2^ IT University of Copenhagen, Copenhagen, Denmark.; ^3^ Department of Psychology and Behavioral Sciences, Aarhus University, Aarhus, Denmark.; ^4^ Laboratory for Telehealth and Telerehabilitation, Center for Sensory-Motor Interaction, Department for Health Science and Technology, Aalborg University, Aalborg, Denmark.; ^5^ Cardiotechnology Laboratory, Medical Informatics, Department for Health Science and Technology, Aalborg University, Aalborg, Denmark.; ^6^ Vendsyssel Hospital, Hjørring, Denmark.

## Abstract

***Objective:*** The aim of this article is to describe the development and testing of a prototype application (“The Heart Game”) using gamification principles to assist heart patients in their telerehabilitation process in the Teledialog project.

***Materials and Methods:*** A prototype game was developed via user-driven innovation and tested on 10 patients 48–89 years of age and their relatives for a period of 2 weeks. The application consisted of a series of daily challenges given to the patients and relatives and was based on several gamification principles. A triangulation of data collection techniques (interviews, participant observations, focus group interviews, and workshop) was used. Interviews with three healthcare professionals and 10 patients were carried out over a period of 2 weeks in order to evaluate the use of the prototype.

***Results:*** The heart patients reported the application to be a useful tool as a part of their telerehabilitation process in everyday life. Gamification and gameful design principles such as leaderboards, relationships, and achievements engaged the patients and relatives. The inclusion of a close relative in the game motivated the patients to perform rehabilitation activities.

***Conclusions:*** “The Heart Game” concept presents a new way to motivate heart patients by using technology as a social and active approach to telerehabilitation. The findings show the potential of using gamification for heart patients as part of a telerehabilitation program. The evaluation indicated that the inclusion of the patient's spouse in the rehabilitation activities could be an effective strategy. A major challenge in using gamification for heart patients is avoiding a sense of defeat while still adjusting the level of difficulty to the individual patient.

## Introduction

Only 13 percent of heart patients participate in rehabilitation activities in Denmark,^1^ and the same low participation rate is seen within rehabilitation of heart patients in Europe and the United States.^2^

Typical problems of heart patients include lack of energy, memory loss, depression, and diminished physical abilities.^3^ The reasons for not engaging in rehabilitation activities are manifold. Among the explanations cited for the low participation rate of heart patients are long transportation times, lack of information about the activities, emotional instability and lack of motivation among the patients, and the time of day due to patients having a job.^4^ Patients who suffer from emotional instability often have difficulties remembering information and taking part in routine rehabilitation activities. A Danish telerehabilitation program, Teledialog,^5^ has been developed and clinically tested in order to identify the needs of heart patients and their family members, prevent re-admission, and design a more individualized rehabilitation program for heart patients through the use of new technologies. A subproject under Teledialog has been to explore whether gamification can be used as a tool to engage heart patients and relatives to perform rehabilitation activities.

Games are a ubiquitous phenomenon in society as well as a growing part of the entertainment industry.^6^ People have access to digital games on their mobile phones, tablets, hand-held gaming devices, computers, and consoles. Mobile and networking technologies create connections between players and offer the opportunity for social and dynamic experiences while also allowing players to access the game independent of time and place.

Games are not only about entertainment. “Gamification” is defined as the use of game design elements in nongame contexts.^7^ The idea is to use elements from games in a vast range of real-life contexts to deal with real issues.^8–13^ Games can be an effective medium to engage and motivate users in order to create behavioral change.^8,14–18^ In a field related to gamification, McGonigal^6^ popularized the term “gameful design,” borrowing ideas from positive psychology.^19^ This project has explored and tested the use of gamification as well as gameful design on rehabilitation of heart patients.

“The Heart Game” prototype applies several successful gamification techniques inspired by findings from literature, such as leaderboards,^17^ point systems,^8,17^ and social aspects like collaboration^18^ and competition.^17^

“The Heart Game” is an application for tablets that was developed in close collaboration with healthcare professionals and patients in the Teledialog project. The application uses gamification principles as a means to motivate heart patients to take an active part in their rehabilitation while also including the patient's spouse in the process. The aim of the project is to assess the potential of gamification as a useful tool for heart patients and their relatives participating in a telerehabilitation program.

This article is based on a Master's thesis project written by Marcus Dithmer and Jack Ord Rasmussen.^20^

## Materials and Methods

### Ethical considerations

The Teledialog study was approved by the local Ethical Committee (approval number 20120051) in August 2012 and performed according to the Helsinki Declaration.^21^

### Target group

The target group consisted of patients diagnosed with heart failure, myocardial infarction, or angina pectoris who were participating in a telerehabilitation program. The patients were in different phases of their rehabilitation process. Although some had only recently started their treatment, others had been out of the hospital for several months and were more physically capable. All patients who evaluated “The Heart Game” had basic knowledge of using a tablet. Table 1 contains an overview of the patients who participated in the study.

**Table 1.** Overview of the Patients Who Participated in the Study

*Patient number*	*Sex*	*Age group (years)*	*Teammate*	*Diagnosis*
1	Male	60–69	Spouse	Myocardial infarction
2	Female	50–59	Spouse	Myocardial infarction
3	Female	70–79	Son	Myocardial infarction
4	Female	50–59	Spouse	Arteriosclerosis, hypertension
5	Male	50–59	Spouse	Myocardial infarction
6	Female	50–59	Other patient	Myocardial infarction
7	Male	60–69	Spouse	Myocardial infarction
8	Male	80–89	Member of project staff	Myocardial infarction
9	Male	40–49	Other patient	Myocardial infarction
10	Male	60–69	Spouse	Arteriosclerosis, hypertension

### User and context studies

The prototype has been developed through a user-driven process,^22^ and a triangulation of data collection techniques has been applied. The data included:

• Qualitative interviews^23^ with two heart patients in their homes. The aim of the interviews was to identify the challenges facing a heart patient following discharge from the hospital.
• Qualitative interviews with two nurses working with heart patients and rehabilitation at two different hospitals to gain their professional perspective on the challenges the patients face.
• Participant observations^24^ at an exercise session for heart patients in order to learn more about the patients' life and treatment. Field notes were taken.
• A workshop with a heart patient and a nurse, the aim of which was to generate ideas for prototypes.
• A focus group interview^24^ with three patients to better understand their everyday lives and discuss some early ideas.

The interviews were carried out by two of the authors, based on semistructured interview guides, lasted 45–60 minutes, and were recorded.

### Development of the prototype

**FIG. 1.**
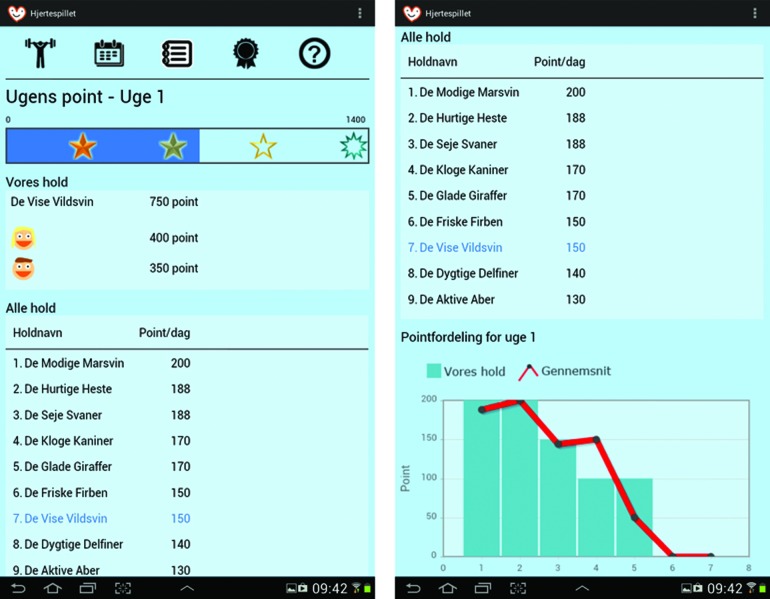
Screen shots of the “Points” page in “The Heart Game” with leaderboard and progress bar. (Color images available at www.liebertonline.com/g4h)

**FIG. 2.**
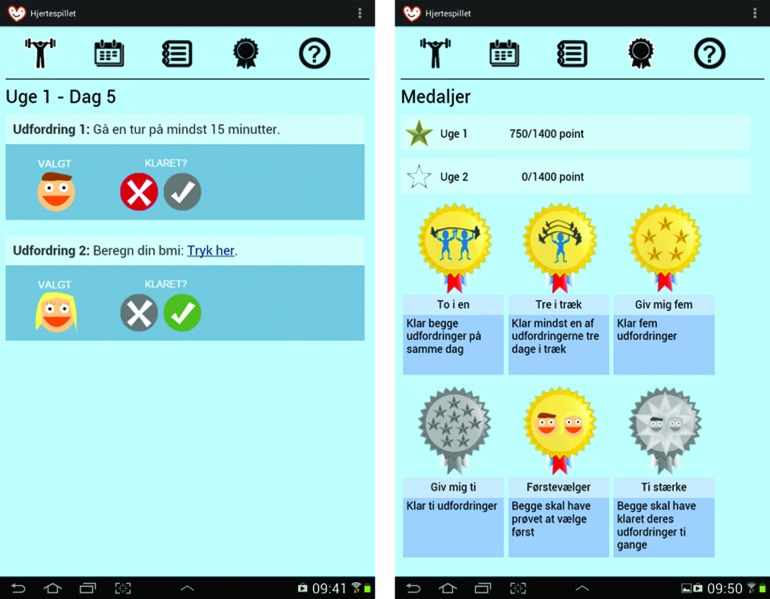
Screen shots of the “Challenges” and “Medals” pages in “The Heart Game.” (Color images available at www.liebertonline.com/g4h)

Based on the user and context studies as well as a review of the literature, a prototype was developed for tablets running the Android™ (Google, Mountain View, CA) operating system. The application presents heart patients with a game-like challenge every day. The game is designed to be played by a two-person team: a patient and someone close to the patient such as a husband or wife. Every day for 2 weeks, the two “players” are presented with two challenges, one for each team member. They each have to choose and carry out one of the challenges. The next day, two new challenges appear, and the completion status of the challenges from the previous day is saved. Some challenges have a link to an exercise or additional information. Examples of challenges include eating a certain amount of fruits or vegetables, going for a walk, dancing, and competing with your teammate in certain tasks. Selecting and completing challenges award points, which accumulate on a weekly basis, to the users. Figures 1 and 2 show screenshots of the application.

The application allows the patient and his or her teammate to view previous challenges along with points earned, points for the week for each of the two players, a weekly progress bar, a leaderboard showing the points earned by other teams playing the game, a graph comparing their team's score with the average score for each day, and medals earned by completing certain tasks, such as reaching a specific number of completed challenges.

**FIG. 3.**
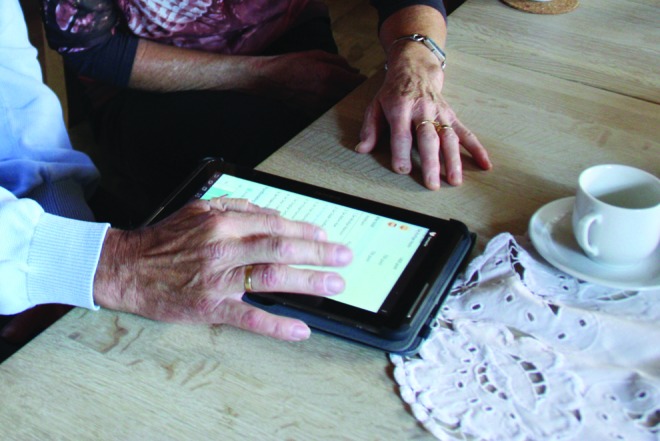
A patient and his wife using “The Heart Game” on a tablet computer. (Color images available at www.liebertonline.com/g4h)

The application is designed to be used soon after discharge from the hospital and when the patient begins the rehabilitation process. Although the application was designed and tested to run on both tablets and smartphones, the evaluation of the prototype was carried out only on tablets (Fig. 3) for the sake of consistency. Ideally, the patients would use their own phones or tablets, but if this is not feasible, a viable option would be for them to borrow an Internet-connected tablet device for the duration of the rehabilitation process. The application was developed as an Android application, with all content accessed in an external database. Ten teams were set up for the evaluation, each with a unique team name.

### Gamification principles

“The Heart Game” uses several gamification principles. These include “badges,” “leaderboards,” and “levels” as defined by, for example, Deterding et al.^7^ Adding the concept of “points” makes up the four principles that form the classic concept of gamification as described by, for example, McGonigal.^25^ Points are given both for selecting and completing challenges. These are shown as a team score on a leaderboard with the other participating teams. An “achievement” system with medals as rewards is used as well. The concept of levels is represented in the form of a progress bar that fills up and displays several stars as the points accumulate.

Zichermann and Cunningham^26^ described a variety of game mechanics, some of which are used in “The Heart Game.” There is a “collection” aspect of obtaining badges and an element of “recognition” through winning them. There is “surprise” every day when the new challenges appear on screen. Finally, the mechanic of “leading others,” which includes team-based cooperative challenges, is the main part of “The Heart Game.” In addition, the mechanic of “feedback” is present through the points given, the progress bar, the badges, and in part the overview of previously completed challenges, which also show points awarded for the separate days.

The four ideas of gameful design are “positive emotion,” “relationships,” “meaning,” and “accomplishment” (abbreviated PERMA).^25^ The first, “positive emotion*,*” is about designing for health, happiness, and well-being. Although these concepts are of a more subjective nature, the main aim of “The Heart Game” is to improve the well-being of the heart patients and to encourage a healthy lifestyle. The second idea, “relationships,” focuses on the husband or wife as a key aspect of “The Heart Game,” while connecting the patient with other teams through the leaderboard lets them know that there are others “out there” working toward the same goals. The relationships aspect also relates to “meaning,” described as giving purpose or setting collective goals. Although the patients are not explicitly presented with a shared goal within the application, they are certainly working toward the same overall goal of completing their rehabilitation and improving their health. The final idea, “accomplishment,” is about achieving something that really matters in everyday life. Health matters to heart patients, and improving their health and returning to normal everyday life after their treatment are worth accomplishing.

### Evaluation

Before the evaluation of “The Heart Game,” the content of the prototype was approved by a cardiologist and nurse from the cardiology ward at Vendsyssel Hospital, Hjørring, Denmark.

“The Heart Game” prototype was evaluated by 10 patients and their spouses/partners for a 2-week period. Table 1 shows the patients and their team partners.

Six patients played the game with a spouse, one patient played the game with her son, two patients played the game with each other remotely (without face-to-face meetings), and one patient played the game with one of the authors because he had no one with which to play. All the teams started the game within 1 day of each other. Two of the 10 patients stopped using the application after 10 days because they left for vacation.

The evaluation of the application was based on the following data collection techniques:

• Log files of the patients' use of the game in order to track their frequency of use and patterns of behavior
• Qualitative interviews with 10 patients and 6 teammates before and after the evaluation period.

The interviews lasted 50–60 minutes. The interviews were conducted by two of the authors using semistructured interview guides and were recorded. The data from interviews were analyzed in steps inspired by the “content analysis” of Kvale and Brinkmann,^23^ where the content is analyzed, condensed, and separated into specific themes.

## Results

Table 2 shows a list of design principles used in “The Heart Game.” The list combines general techniques as well as several gamification and game design principles. Findings in relation to these principles are detailed below.

**Table 2.** Design Principles Used in “The Heart Game”

*Design principle*	*Implementation*	*The experiences of the patients*
Daily challenges	Daily revealing of next challenges	The challenges were completed every day by all 10 patients.
Leaderboard	Ranking of teams by points	Nine out of the 10 patients reported being motivated by this.
Points	Points given for selecting and completing challenges	Placement was more important than points, and maximum possible points were used by patients to measure performance.
Achievements	Medals for specific goals	Too hidden in the user interface. Three out of the 10 patients wanted to collect all medals.
Surprise mechanic	New challenges every day	All 10 patients expressed motivation by this feature, and 1 patient stated “It is like an Advent calendar.”
Including the spouse/family member	Spouse as teammate	Nine out of the 10 patients expressed that it helped them to complete tasks and that there being two persons encouraged them.
Variation	Different types of challenges	Eight of the 10 patients liked the challenges, and 2 of the 10 asked for more challenges.
Meaning	Relevant rehabilitation tasks	The tasks were not just seen as a game and had value for their rehabilitation and helping them return to everyday life.
Collective goal	Patients compete against each other.	Promoted completion and social networking among the patients

Patients include the two teams that stopped after 10 days.

Of the 20 players (10 patients and 10 partners), 18 stated that they enjoyed playing the game. They said it was a fun and novel way to approach rehabilitation and to deal with the issues they were having. “The Heart Game” acted as a daily reminder to all the patients of certain exercises they had to do and lifestyle changes they needed to make. For example, two teams explicitly stated that on a day with bad weather, they would not have gone for a walk had they not been motivated by the application.

Playing as a team enabled the relative to play a bigger part in the patient's rehabilitation program. Nine out of 10 teams liked playing with a partner, and some found it to be the most important aspect of “The Heart Game.”

The log data revealed that all teams had used the application daily. The main part of the game, completing the challenges, was used every day during the evaluation, and the leaderboard was viewed slightly less, generally every third day. All but one team stated that the leaderboard and score had motivated them “to some degree” to complete the challenges presented by the application. The score was used to compare themselves with other teams and to see how well they were progressing. Most teams cared more about their position relative to other teams than their precise number of points.

The achievements, in the form of medals, were viewed a few times by all teams, but not on a daily basis. This accords with the responses in the interviews, as all teams said they did not find the medals as engaging as the points. Several teams found the medals to be too hidden, and it was unclear exactly when they won any. However, they did know what they needed to do to earn them, and a few teams mentioned actively trying to earn medals, indicating that this recognition meant something to them. In addition, a few of the teams actively tried to collect the entire set of medals.

The general mechanic of feedback was found to be valuable to heart patients because they could see their progress when completing the challenges.

The surprise mechanic of seeing new challenges every day was very positively received. Using unknown elements, however, should be carefully considered if they are being used in the early stages of rehabilitation because the patients are likely in a fragile state. The idea of using cooperative challenges was shown to be an effective motivator for the patients. Two teams expressed a wish for intellectual challenges such as crossword puzzles, Sudoku, or memory games. Another team would have enjoyed some challenges with focus on psychological aspects. The more light-hearted challenges such as laughing out loud and dancing were generally well received.

## Discussion

The prototyping and evaluation done in this project are of an exploratory nature, and thus the results should be viewed as such. All interviews were not conducted by the same person, and content analysis was carried out by the authors, which could potentially have affected the reliability of the data. A randomized controlled trial would be needed to assess whether “The Heart Game” is an effective tool for helping heart patients in their rehabilitation process, as this initial testing and evaluation seem to indicate.

It was found that the patients enjoyed using “The Heart Game” while they carried out the challenges and that they believed it to be useful in their rehabilitation. It is also worth noting that not only was “The Heart Game” generally seen by the patients as a positive addition to the current rehabilitation process, but the idea of using a gamified application on a tablet was also well received.

The use of points and leaderboards in the “MoviPill”^17^ prototype to improve medication adherence is similar to that of “The Heart Game,” and here the social competition aspect was also a motivating factor. The developers of the “Playful Bottle”^18^ prototype concluded that reminders from other players are more effective than system notifications. “The Heart Game” uses system notifications, but it has the potential to create more communication between the teams, a conclusion also suggested by the patients and their spouses.

An abstracted version of points can be seen in prototype games such as the “Playful Bottle”^18^ and “Fish'n'Steps,”^8^ where withering trees and crying fish were successfully applied as scores. This kind of abstraction was not explored in “The Heart Game,” but based on user studies of the heart patients, a mechanic such as nurturing the fish could prove challenging to implement if the patients feel too upset if the fish dies and they feel they have failed.

A randomized controlled trial of the “SuperBetter”^11^ application, which was used by people suffering from depression, showed positive results. One of the techniques mirrored in “The Heart Game” was that of creating small, manageable tasks—something positively received by the heart patients. It seems that some aspects of the rehabilitation process are more suited for forms of gamification than others. The heart patients are physically and mentally unstable immediately following heart surgery, and in this first period of the rehabilitation, some aspects of gamification that require too much effort or commitment from the patient, such as physical challenges and competitions, might best be modified or applied with great care. The risk of losing could cause increased stress for the patient.

A limitation of the study was that the patients were in different phases of their rehabilitation process. Some had only recently begun treatment, whereas others had gone several months since their hospital admittance. This contrast became apparent in the evaluation, as most teams found the challenges too easy because they had already completed them as part of their daily rehabilitative regimen.

A challenge for heart rehabilitation and health care in general is the lack of individually tailored programs for the patients. “The Heart Game” and the use of mobile applications and gamification generally make it easier for the patients to adjust their rehabilitation needs because the mobile application offers a higher degree of flexibility with regard to time and place. A need for greater personalization was identified, as the skill level and overall health of the patients varied. This could potentially be achieved by establishing a leveling system, so the difficulty would match the capabilities of the individual patient and be integrated as part of the patient's overall rehabilitation plan.

The timing of use of “The Heart Game” also relates to other challenges: determining when in the rehabilitation process “The Heart Game” is most useful to the patients, as well as how long the game should run. All patients agreed that it should not be used for less than 2 weeks, and most suggested that it should run for longer, up to a period of 10–12 weeks, which would ideally require more challenges. The initial interest in “Fish'n'Steps”^8^ faded after a few weeks, which is worth noting in regard to “The Heart Game,” as this was only evaluated for 2 weeks in total.

The four pillars of gameful design (PERMA) were found to be very relevant. Being more subjective than the more traditional principles of gamification, they are also more difficult to implement. A very important aspect of the rehabilitation is for the heart patients to have support during the process.^3^ One way of creating positive emotions for the patients is to include a close relative, hence the importance of “relationships” as part of the PERMA framework. All teams but one expressed satisfaction in being able to play the game with a partner. Some of the patients found the most important aspect of “The Heart Game” was the inclusion of their relative, enabling them to deal with their rehabilitation as a team. Several teams competed in a friendly manner when choosing challenges or by giving each other the toughest one.

Gamification and the use of mobile technologies are currently being explored and are said to hold great potential for future healthcare solutions.^27^ The exploration carried out in this article highlights gamification as a promising platform for use in the rehabilitation process of heart patients. Being motivated is a major factor, and gamification is a useful tool to increase motivation. Looking at the healthcare sector as a whole, gamification offers possibilities that go beyond the heart rehabilitation process discussed in this article. Other rehabilitation processes involving lifestyle changes and motivation are ideal candidates for gamification. Gamification focuses on making necessary and tiresome tasks more enjoyable. As a means of enhancing motivation, gamification can potentially assist patients in those areas of health care where patients need to deal with chronic diseases or disabilities.
